# Management of Myofascial Pain of Upper Trapezius: A Three Group Comparison Study

**DOI:** 10.5539/gjhs.v4n5p46

**Published:** 2012-07-15

**Authors:** Priya Kannan

**Affiliations:** 1 Post Graduate student, Centre for Physiotherapy Research, University of Otago, New Zealand

**Keywords:** laser, ultrasound, ischemic compression, myofascial trigger point

## Abstract

It is important to identify the most effective therapeutic modality in the management of myofascial trigger points (MTPt). Thus we aimed to study the effect of therapeutic ultrasound, laser and ischemic compression in reducing pain and improving cervical range of motion among patients with MTPt. Experimental study comparing three groups was designed as a 5 days trial, a co-relational design was considered. Outcome measures: VAS for pain, provocative pain test using “soft tissue tenderness grading scheme” and active cervical lateral flexion using inch tape. Methods- Patients were divided into 3 groups, Gr 1 underwent treatment using therapeutic ultrasound, Gr 2 with therapeutic laserand Gr 3 with ischemic compression. Assessments were done on day 1 and day 5 of treatment respectively. Results: ANOVA revealed improvement among all 3 groups as statistically significant difference (p<0.05) between the start and end of trial. Analysis using Chi square test shows a statistically significant difference in the improvement between laser and the other 2 groups. Mean difference in the change of scores between the assessments showed laser therapy to have a tendency towards progressive improvement over the treatment period and a better improvement than the other 2 groups. Weconclude that laser can be used as an effective treatment regimen in the management of myofascial trigger points thereby reducing disability caused due to musculoskeletal pathology.

## 1. Introduction

Myofascial trigger point (MTPt) can be defined as a hyperirritable spot in skeletal muscle that is associated with a hypersensitive palpable nodule in a taut band (Lois, 1999). MTPt is associated with pain on compression and the pain is typically of a referred type. MTPt are among the most common, yet poorly recognized and inadequately managed causes of myofascial pain seen in medical practice with a point of prevalence from 10% to 18% and lifetime prevalence from 30% to 50% (Çakıt, 2009). In many people with MTPt symptoms, cause severe discomfort and inability to work. Myofascial pain symptoms usually involve muscle pain with specific “trigger” or “tender” points. The pain is aggravated with activity or stress. In addition to the local or regional pain, untreated and chronic cases might lead to symptoms like depression, fatigue and behavioral disturbances.

The causes for myofascialpain are structural inadequacies, tight constrictive clothing, systemic, alcohol toxicity, inflammatory diseases and relative growth hormone deficiency. There are various treatment modalities used for treating myofascial pain which includes individual treatment techniques under manual therapy, acupuncture, stress reduction, electrotherapy, body mechanics & ergonomic training, nutritional counselling and a wide range of pharmacological management (Borg-Stein, 2002).

Ultrasound (US) treatment is one of the most important physical treatment modalities in MTPt treatment which is used for heating deep tissues. It is a noninvasive method which consists of piezoelectric crystals that convert the electrical energy to mechanical oscillation energy using high-frequency alternating current (van der Windt, 1999). US increase local metabolism, circulation, regeneration and extensibility of connective tissue with its assuming thermal and mechanical effects. However, results in the studies related to its efficacy in the musculoskeletal system problems are conflicting (Gam, 1998; Robertson, 2001).

Due to the effectiveness of laser in treating many types of conditions, and the short treatment time required, laser has been widely used as a complementary therapy, and hence it has been used in combination with other Physiotherapeutic modalities. However, laser is rated high in its effectiveness than other conventional modalities in literature. There are evidence to show the effect of laser therapy in the management of myofascial pain, however the results appear to be variable based on the parameters used for treatment (Al, 2009).

MTPt is treated with “ischemic compression” by applying heavy thumb pressure on painful spot, sufficient to produce skin blanching (Simon, 1999). They can also be treated by applying gentle digital pressure to the area. This fundamental change is anchored in Travel’s ATP energy crisis model, which characterizes MTPt as centres of tissue hypoxia. Thus, deep tissue pressure that produces additional ischemia is not beneficial. Alternatively a “press and stretch” method restores the normal resting length of the sarcomere through barrier release concept where the finger follows the releasing tissue.

There seems to be a lack in the literature to establish the effectiveness of laser when compared to ischemic compression and therapeutic ultrasound in the treatment of MTPt of upper trapezius muscle. There is a need for a comparative study on the effect of the above mentioned therapeutic modalities. Thus the current study is aimed at evaluating:

- The pain relief produced by laser, therapeutic ultrasound and ischemic compression in treatment of MTPt’s.
- The effect of laser, therapeutic ultrasound and ischemic compression on the lateral flexion of the neck.
- The effect of laser, therapeutic ultrasound and ischemic compression on the tenderness due to MTPt.

## 2. Materials and Methods

Patients with cervical and upper trapezius myofascial pain were recruited from outpatient service, Masterskill Physiotherapy and Research Centre (MSPRC), Malaysia. Ethical clearance from the institute research board was obtained.

### 2.1 Patient Selection

**Figure 1:**
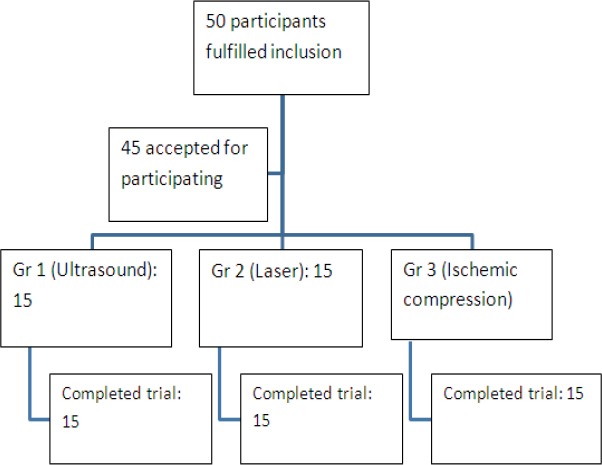
Study flow chart

Patients with clinically active, palpable MTPt on one side or both sides of the upper trapezius muscle were recruited. The criteria for excluding subjects were (i) patients with CNS deficits, (ii) patients with cognitive deficits, (iii) patients receiving treatment by other methods like vapocoolants, dry needling, acupuncture injections etc, (iv) patients with MTPt in muscles other than trapezius and (v) patients with hypermobile joints. A total of 50 patients fell under inclusion criteria, 45 consented for participation (22 women, 23 men; age range, 20–40yrs). 15 subjects were allotted into each group randomly and all the subjects were available for the post test assessment as shown in Figure 1.

### 2.2 Study Design

Thisquantitative research analysis was designed as a 5 days trial, a multi group pretest posttest design was considered.

### 2.3 Outcome Measures

Measurement of subjective pain was assessed using a visual analog scale (VAS), provocative pain test using “soft tissue tenderness grading scheme” and active lateral bending of the cervical spine using inch tape were done before the first sessions and after five consecutive sessions of therapy.

VAS was used to evaluate and quantify the perceived pain by the subjects. Origin of the scale is indicated as “NO PAIN” and the terminal end as “MOST SEVERE PAIN”. The patient was instructed to move the indicator to represent his/her pain perceived. At the back of the scale 0 to 10 numerical with a distance of 1cm between them were marked. The linear analogue rating of the constant pain stimulus is reproducible and changes in rating are likely to be real change in opinion.

The “Tenderness grading scale” (Hubbard, 1993) is a proposed grading system for the soft tissue tenderness. It is also a method for documenting patient responses to “provocative” tests, such as orthopedic tests or the McKenzie analysis. Tenderness grading is as follows:

0- No tenderness
1- Tenderness to palpation without grimace or flinch
2- Tenderness with grimace & or flinch to palpation
3- Tenderness with withdrawal (+ “ Jump sign”)
4- Withdrawal (+ “Jump sign”) to non-noxious stimuli (i.e. superficial palpation, pin prick, gentle percussion)

Tape measurement: tool used is a simple inch tape. Patient was positioned in relaxed high sitting with backrest, head and neck in neutral position arms resting on the thigh or a pillow on the lap, measurement was taken from the mastoid process to the same side acromion process and the initial reading was recorded. Then the patient was asked to laterally flex his/her neck to the opposite side as far as possible with the therapist holding the inch tape fixed to the mastoid process end fixed and allowing the acromion end to move freely. At the end of maximum lateral flexion the final reading was recorded. The range of motion was calculated as the difference between the neutral reading and the reading in maximum lateral flexion.

### 2.4 Procedure

Patients were divided into 3 groups according to a simple randomization scheme^9^. The first group (Gr 1) was treated using therapeutic ultrasound, treatment given over the most tender spot, second group (Gr 2) with therapeutic laser and third group (Gr 3) with ischemic compression. Upper trapezius stretch was advised as home program for patients of all 3 groups. The treatment was given for five consecutive days: Initial assessment was done on day 1 prior to the therapy and the final assessment on day 5. Patients were informed that they would be subjectto one of three possible different treatment procedures in order to evaluate the most effective treatment for pain. All patients were evaluated by the same examiner who was blinded regarding the treatment. A written consent was obtained before the start of the trial. The treatments were performed by the principal examiner. The values obtained were compared and analyzed to study the pain relief produced by ischemic compression, therapeutic laser and therapeutic high power ultrasound.

### 2.5 Treatment Parameter

Therapeutic ultrasound: U/S head size- 1cm, mode- continuous, Intensity- variable according to pain threshold but within 1.5 watts/cm^2^, Range- 0.1 to 1.5 watts/ cm^2^, Treatment time- 5 mins and patient position- high sitting with back rest.

Therapeutic laser: probe size- point probe 1cm diameter, Wave length- 904nm, mode- Pulsed, Dose- 74mJ/cm^2^, Penetration depth- 1cm, Treatment time- 30sec and patient position- High sitting with back rest.

Ischemic compression: manual digital pressure, treatment time- 5mins, patient position- supine lying with small pillow ora towel roll to support the neck. Treatment technique- Initially thumb (or strong finger) was pressed directly on the trigger point to create tolerable painful, sustained pressure. Pressure was gradually increased by adding a thumb or finger from the other hand as necessary for reinforcement. This pressure was continued up to one and a half minute with as much as 20 to 30 lb of pressure. If the trigger point tenderness persisted, the procedure was repeated. Treatment was considered useless if the patient tend to abate.

### 2.6 Statistical Analysis

The results of statistical analysis were expressed as mean ± SD (Standard deviations) (Wilson, 1997). To compare the differences within the groups, ANOVA was used. The Chi square test was used to calculate the differences between the outcome variables between the groups (Gr 2 against Gr 1 and Gr 3) along the study period. A significance level of p value less than 0.05 was used for all comparisons. All analysis was performed using SPSS software.

## 3. Results and Discussion

**Figure 2:**
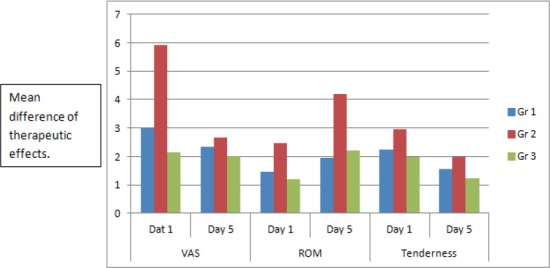
Comparing the mean difference between start and end of trial among the 3 groups

There were no dropouts from the study. No differences existed between the groups in terms of age and gender. Demographics were explained as illustrated in table 1. Within group analysis using ANOVA revealed a statistically significant (p< 0.05) difference among all the three groups between pre test and post test scores. There was an insignificant (p= 0.65) difference between pre test and post test scores of tenderness grading among all three groups. The mean difference in the change of score of the variables among the three groups were plotted on a graph which showed Gr 2 to have a higher tendency of change between the start and end of the trial, as shown in figure 2. Chi square was used to compare the difference seen in Gr 2 against Gr 1 and Gr 3. It was found that there was a statistically significant difference (p< 0.05) between the change of score in Gr 2 when compared to the other two groups as shown in table 3.

**Table 1:** Demographic and baseline characteristics of the 3 groups and the total subjects at the start of the study

Characteristics	Gr 1(n=15)	Gr 2 (n=15)	Gr 3 (n=15)	Total (n=45)
Age (Mean ± SD)	32±9.33	29±10.23	31.24±9.34	30.67±10.22
Sex (M:F)	7:8	5:10	11:4	23:22
VAS (Mean)	3.00	5.93	2.13	4.33
ROM (Mean) inches	1.46	2.46	1.20	2.12
Tenderness (Mean)	2.23	2.96	1.97	1.45

VAS: Visual analogue scale ROM: Range of motion Gr 1: Ultrasound Gr 2: Laser and Gr 3: Ischemic compression

**Table 2:** Within group analysis outcomes between day 1 (pre-test) and day 5 (post-test)

Variables	Assessment	Treatment groups			ANOVA	

		Gr 1	Gr 2	Gr 3	F	p
VAS	Mean ± SD of day1	3.00±0.22	5.93±1.33	2.13±0.96	2.11	0.003*
	Mean ± SD of day5	2.34±0.45	2.66±1.23	2.02±0.88	1.96	
ROM	Mean ± SD of day1	1.46±0.78	2.46±1.22	1.20±1.12	2.21	0.001*
	Mean ± SD of day5	1.96±0.78	4.21±1.23	2.20±1.42	3.21	
Tenderness	Mean ± SD of day1	2.23±0.89	2.96±0.78	1.97±1.12	2.54	0.65
	Mean ± SD of day5	1.56±0.98	1.97±0.98	1.22±0.98	2.43	

VAS: Visual analogue scale ROM: Range of motion Gr 1: Ultrasound Gr 2: Laser and Gr 3: Ischemic compression

* Statistically significant

**Table 3:** Comparison of the testing variables of Gr 2 against Gr 1 and Gr 3 Chi square test

Outcomes	Gr 1 (mean± SD)	Gr 3 (mean± SD)	Chi- square	Significance
Comparison of VAS (Gr 2 against Gr 1 and Gr 3)	1.12±0.60	0.73±0.74	24.94	0.000
Comparison of ROM (Gr 2 against Gr 1 and Gr 3)	1.16±0.33	0.29±0.94	10.73	0.005
Comparison of Tenderness (Gr 2 against Gr 1 and Gr 3)	1. 20 ±0.56	0.26±0.59	21.83	0.000

VAS: Visual analogue scale ROM: Range of motion Gr 1: Ultrasound Gr 2: Laser and Gr 3: Ischemic compression

The main issue in the MTPt treatment is to provide pain relief on trigger points. The major treatment methods are patient training, elimination of trigger factors, medical treatment, superficial & deep heat applications, electrotherapy, stretching and spray technique, acupuncture, local injections, massage and exercise (Byl, 1995; Kozanoglu, 2003). The current trial was designed as a three group comparative analysis to objectively record the effect of therapeutic ultrasound, laser and ischemic compression in improving ROM & reducing pain among cases with MTPt. The age restriction of 20-40 years minimized the influence of pain from degenerative joint and disc disease. The provision of information on environmental perpetuating factors to patients in the study enhanced the possible therapeutic influence of the therapeutic modalities to be tested. Evaluation of the data revealed that all three groups showed a significant decrease in the levels of pain perception, overall pain intensity and extent to which the patients were suffering with myofascial pain syndrome together with an overall increase in cervical lateral flexion range. Analysing further the results of the present study show the possible short-term therapeutic effects of laser in the treatment of MTPt as measured by both subjective and objective indices. Analysis using Chi square revealed that there is a statistically significant improvement in all testing variables among subjects of Gr 2 when compared to the other two groups.

The use of High power ultra sound is recommended as clinical therapy for chronic MTPt (Esenyel, 2000; Majlesi, 2004). In the literatures that support the use of ultrasound it was found that the pain relief was due to its assuming thermal and mechanical effect. Draper Do et al, in his study has put forward the beneficial effect of thermal ultrasound and has stated that the thermal ultrasound technique over latent trigger points is comfortable and can decrease stiffness of a trigger point (Draper, 2010). However, in another RCT, Gam et al found no difference between groups given conventional ultrasound or sham ultrasound in the treatment of myofascial trigger points in the neck and shoulder (Gam, 1998). This trial has shown evidence for the positive effect of therapeutic ultrasound in improving lateral flexion of neck and a reduction in the perceived level of pain.

Laser has a wide spectrum of applications in medicine. Clinical studies have shown Laser to be as effective as analgesics and to accelerate the healing of injured tissue (Dundar, 2007). Laser seemed to have the best effect if the patient was informed about the treatment and the appliance was activated (Katsoulis, 2010). Researchers have suggested mechanisms for pain relief using laser, which includes the secretion of endogenous opioids, such as in acupuncture and transcutaneous electrical nerve stimulation, leading to clearance of the analgesic substances via stimulation of the microcirculatory system (Brosseau, 2000; Lee, 1996). In our study we observed a significant difference in the outcome measures between the start and end of the trial. Figure 2 illustrates the difference between the 3 groups to be superior among the Gr2 patients. The more sustained improvement seen in the Gr2 may be attributed to the therapeutically significant benefit of Laser’s ability to stimulate protein synthesis, soft tissue repair and subsequent tissue regeneration.

Pain relief from ischemic compression treatment may have resulted from reactive hyperaemia in the MTPt region (Simon, 1999), counter-irritant effects (Patrick, 1984), or a spinal reflex mechanism for the relief of muscle spasm. Pressure that is applied to the MTPt of taut band should be within a tolerable pain level for each patient to avoid causing excessive pain and autonomic responses with involuntary muscle tensing. The treatment may not be effective if insufficient pressure is applied. Therefore, an appropriate pressure was used to treat our patients and in addition all patients were treated by the same researcher which aided us to reduce bias due to faulty treatment.

Thus it is evident that all three types of therapeutic modalities may have a similar, but not identical mechanism of action at neuromuscular level. Indeed, laser therapy has shown a superior increment in the study variables when compared to ultrasound and ischemic compression. Though there are literatures which support the influence of the ultrasound and ischemic compression in the management of MTPt we were not able to explain why these techniques did not elicit an outcome similar to Laser. In our observation ultra sound and ischemic compression technique requires additional training and experience for the treating therapist. It requires good communication and concentration for both patient and therapist. Whereas Laser has the advantage over these factors which makes laser a handy tool in the management of MTPts. There were few limitations in our study which includes lack of a control group, limited study sample and short term follow up of therapeutic benefits. This study could be repeated with an additional group undergoing placebo therapy to rule out the fact that pain relief could have been due to time factor.

## 4. Conclusion

Perceived pain and cervical ROM has showed a consistent rise on the subject who were treated using Laser. This is an apparent indication for pain relief caused in the management of MTPt. Treatment efficiency and ease of administration of this technique ensures its frequent usage by clinical practitioners. Thus we conclude stating laser can be used as an effective treatment regimen in the management of myofascial trigger points thereby reducing disability caused due to the musculoskeletal pathology.
